# Anomalies and Curiosities of Medicine

**Published:** 1898-06

**Authors:** 


ANOMALIES AND CURIOSITIES OF MEDICINE. Being an Encyclopedia Collection of Rare and Extraordinary Cases, and of the Most Striking Instances of Abnormality in all Branches of Medicine and Surgery, Derived from an Exhaustive Research of Medical Literature from its Origin to the Present Day. Abstracted, Classified, Annotated and Indexed. By Geo. M. Gould, A. M., M. D., and Walter L. Pyle, A. M., M. D. Fully Illustrated. Published by W. B. Saunders, Philadelphia. 1897. 968 pages. Price by subscription only, cloth, $6. Half Morrocco, $7.

The names of Gould and Pyle are a sufficient guarantee that this work is everything that its title page would indicate. All the museums of the world do not contain as many quaint curiosities. To say that it is the physicians' wonderland is to put it mildly. Of course the distinguished authors have not written for the sake of creating a sensation; on the contrary, every statement made bears evidence of care, and strict intention of veracity. Cases are detailed of women who have menstruated from the breasts, from cicatrices on the hands, from the eyes, and from cicatrices on the lower extremities. Men have menstruated from the penis and urethra, and other situations, and have suffered from amenorrhœa.

Unique cases of accidental conception, and superfœtation, extrauterine pregnancies, gestations phenomenally long and short, and simulated pregnancies, are described.

There are strange cases of maternal and of paternal impressions and telegony. All kinds of human monsters, headless, armless, legless, as well as those who have had more than the usual complement of these useful organs, are described and illustrated.

Numerous cases of such anatomical curiosities as dermoid cysts, pseudo-hermaphrodism and many others of equal scientific interest are treated of.

In conclusion it suffices to state that no adequate description of the contents of this unique work can be given in the brief space alloted to this review. "Anomalies and Curiosities" by Gould and Pyle will become a classic. J.

